# *BCORL1* S878G, *GNB1* G116S, *SH2B3* A536T, and *KMT2D* S3708R tetramutation co-contribute to a pediatric acute myeloid leukemia: Case report and literature review

**DOI:** 10.3389/fped.2022.993952

**Published:** 2022-10-17

**Authors:** Liang Wang, Sen Chen, Yongming Shen, Ping Si

**Affiliations:** ^1^ Department of Clinical Laboratory, Tianjin Children’s Hospital/Children's Hospital of Tianjin University, Tianjin, China; ^2^ Department of Hematology, Tianjin Children’s Hospital/Children's Hospital of Tianjin University, Tianjin, China

**Keywords:** acute myeloid leukemia, *BCORL1*, *GNB1*, *SH2B3*, *KMT2D*

## Abstract

Acute myeloid leukemia (AML) is a clinically, morphologically, and genetically heterogeneous group of malignancies characterized by a wide range of genomic alterations responsible for defective regulation of the differentiation and self-renewal programs of hematopoietic stem cells. Here, we report a 4-month-old boy who had acute onset with leukocytosis and abdominal mass. The morphological analysis of bone marrow (BM) smear revealed extremely marrow hyperplasia, large quantities of immature cells, and primary and immature monocytic hyperplasia accounting for 57.5% of nucleated cells. The chromosome karyotype of the case was complex, representing 48, XY, +13, +19[12]/48, idem, del (p12)[8]. After RNAs sequencing, a mutation (c.346G > A, p.G116S) of the *GNB1* gene was detected and localized to the mutational hotspot in Exon 7. Meanwhile, the other three mutations were identified by next-generation sequencing (NGS) and whole-exome sequencing (WES) of DNA from the BM aspirate and oral swab, including *BCORL1* mutation [c.2632A > G, p.S878G, mutation allele frequency (VAF): 99.95%], *SH2B3* mutation (c.1606G > A, p.A536T, VAF: 51.17%), and *KMT2D* mutation (c.11124C > G, p.S3708R, VAF: 48.95%). *BCORL1* mutations have been associated with the pathogenesis of AML, whereas other mutations have rarely been previously reported in pediatric AML. The patient did not undergo the combination chemotherapy and eventually died of respiratory failure. In conclusion, the concurrence of *BCORL1*, *GNB1*, *SH2B3,* and *KMT2D* mutations may be a mutationally detrimental combination and contribute to disease progression.

## Introduction

Acute myeloid leukemia (AML) is a complex and dynamic disease characterized by high molecular heterogeneity, representing 15%–20% of leukemias in children (1). The development of cytogenetic and molecular biological techniques in recent years has significantly contributed to a better understanding of the molecular and genomic landscapes of AML. A number of genetic mutations have been identified in pediatric AML, which are related to biological, clinical, and prognostic implications. These genes included *RUNX1*, *NPM1*, *CEBPA*, *FLT3*, *MYST3*, *EVI1*, *KIT*, *IDH1* and *IDH2*, *DDX41*, *ETV6*, *GATA2*, *TP53,* and *BCOR*/*BCORL1* (2, 3). The BCL6 corepressor (*BCOR*) gene, and its homolog, the BCL6 corepressor-like 1 (*BCORL1*) mutations, mostly represented frameshifts (deletions, insertions), nonsense and missense mutations, which led to the lack or low expression of the protein. *BCORL1* mutations were detected in 3.7%–5.8% of AML adult patients and 1.2% of pediatric AML patients (4).

Mutations in guanine nucleotide-binding protein (G protein), and beta polypeptide 1 (*GNB1*) have been linked to neurodevelopmental syndrome (5). SH2B adaptor protein 3 (*SH2B3*) mutations have been identified in a range of hematological diseases, including myeloproliferative neoplasms (MPN), myelodysplastic syndromes (MDS), MDS/MPN overlap syndromes, and acute lymphoblastic leukemia (ALL) (6). Mutations in lysine-specific methyltransferase 2D (*KTM2D*) were the predominant cause of diffuse large B-cell lymphoma and follicular lymphoma (7). *GNB1*, *KMT2D,* and *SH2B3* mutations were rarely described in pediatric AML. We reported a pediatric AML, while rare, bearing *BCORL1*, *GNB1*, *KMT2D,* and *SH2B3* tetramutation and reviewed the relevant literature and cases of AML to improve the understanding of this rare form of pediatric AML.

## Case report

### Case presentation

A 4-month-old boy presented to the hospital in June 2021 with a high-grade fever lasting more than 6 days and diarrhea, and a CBC showing anemia (HGB 97 g/L) and leukocytosis (WBC 91.57 × 10^9^/L). Physical examination upon admission showed an absence of spleen/liver enlargement or cutaneous lesions but the existence of abdominal mass. A chest x-Rry was performed, showing the inflammatory consolidation of the lungs. The patient was admitted to the hospital with a suspicion of leukemia. After admission, follow-up blood tests confirmed the presence of moderate anemia (HGB 83 g/L, MCV 86.7 fl, MCH 26.9 pg, MCHC 311 g/L) and leukocytosis (WBC 116.44 × 10^9^/L) with 60% blasts on the peripheral blood smear, as well as the elevated d-dimer (1.11 mg/L), Cr (59 µmol/L), UA (487 µmol/L), GGT (788 U/L), AST (111 U/L), LDH (1,830 U/L), and CRP (19.8 mg/L). In addition, blood, urine, and stool culture were all negative. The examinations of the cerebrospinal fluid showed no significant abnormalities. An examination of the patient’s bone marrow (BM) smear revealed extremely marrow hyperplasia, large quantities of immature cells, primary and immature monocytic hyperplasia accounting for 57.5% of 200 nucleated cells, hypoplasia in the granulocyte and erythrocyte series [granulocyte series accounting for 25.5% (reference range: 40%–60%) and erythrocyte series accounting for 3% (reference range: 15%–25%)] and lymphopenia. Immunohistochemistry showed: leukemic cells HLA-DR(+), CD4(+), CD7(+), CD33(+), CD34(+), CD117(+), CD123(+). The immunophenotyping and morphological analysis on BM aspirate showed suggestive AML-M5.

### Genetic analysis

**Figure 1:**
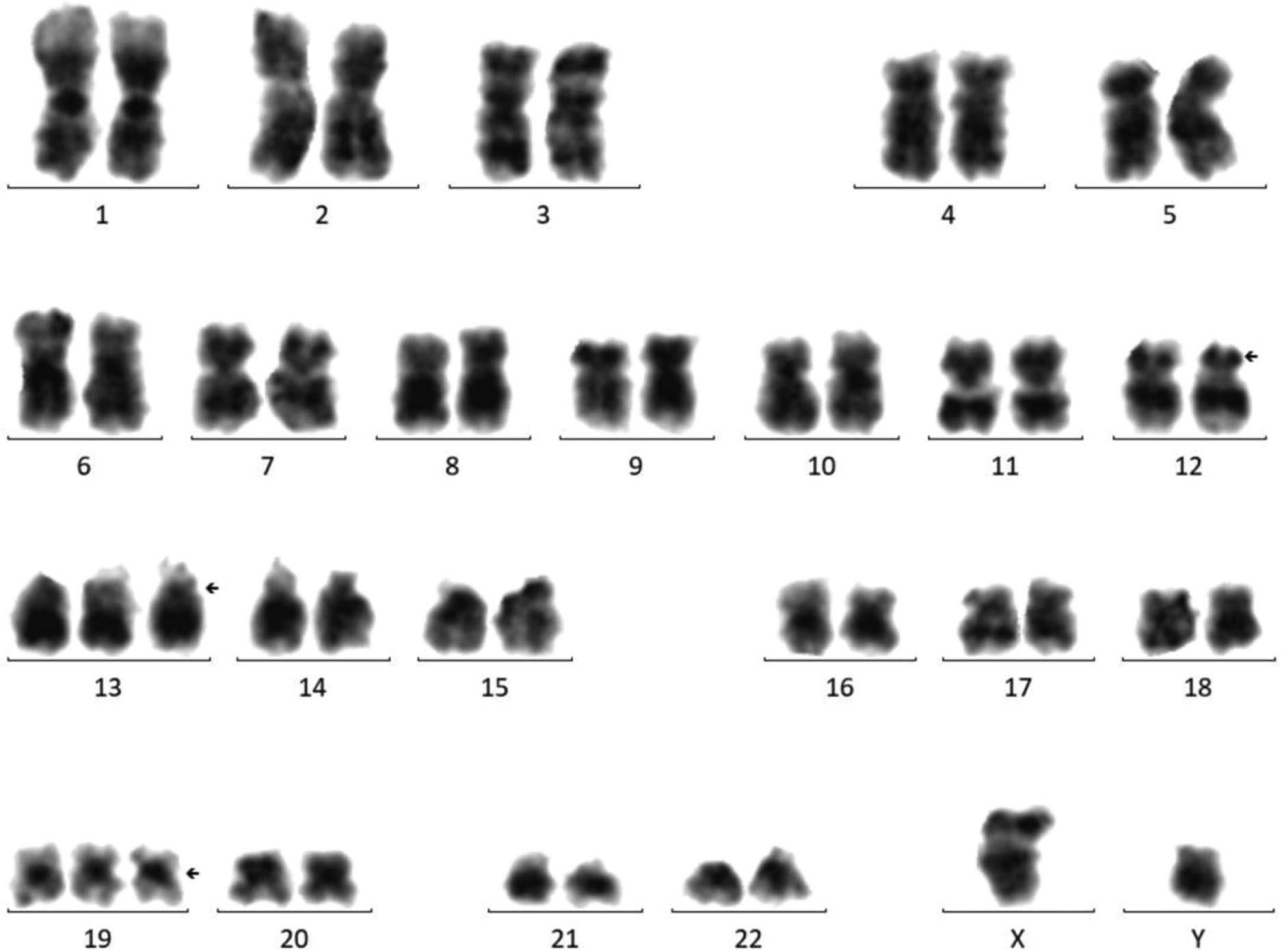
Bone marrow karyotype showing 48, XY, +13, +19[12]/48, idem, del(p12)[8].

Cytogenetic analysis revealed that the chromosome karyotype was complex, representing 48, XY, +13, +19[12]/48, idem, del(p12)[8] (Figure 1). FISH studies and multiplex leukemia translocation assay were negative for common fusion translocations in AML. After RNAs sequencing was performed with the Illumina HiSeq X platform, a mutation (p.G116S) of the *GNB1* gene was detected, which localized to the mutational hotspot in Exon 7. The clinical and/or experimental evidence of genomic variants were assessed by the Association for Molecular Pathology (AMP), American Society of Clinical Oncology (ASCO), and College of American Pathologists (CAP) proposed Standards and Guidelines for the Interpretation and Reporting of Sequence Variants in Cancer (8). Sequence variants were categorized into four categories based on their clinical impact: tier I, variants with strong clinical significance (level A and B evidence); tier II, variants with potential clinical significance (level C or D evidence); tier III, variants with unknown clinical significance; and tier IV, variants that are benign or likely benign. The variant of GNB1 (G116S) in this report was categorized into tier II, which may be associated with the pathogenesis of AML.

Furthermore, a next-generation sequencing (NGS)-based analysis and whole-exome sequencing (WES) for the detection of mutations were performed on the DNAs extracted from BM samples and oral swabs containing exfoliated cells. The three mutations were detected, including *BCORL1* mutation (p.S878G), *SH2B3* mutation (p.A536T), and *KMT2D* mutation (p.S3708R), and it was confirmed that these mutations were germline variants (Table 1). We determined the pathogenicity of our variants *via* the rules for combining criteria to classify sequence variants claimed in 2015 the American College of Medical Genetics and Genomics (ACMG) and AMP proposed Standards and Guidelines for the Interpretation of Sequence Variants (2015 ACMG/AMP guidelines) (9). Variants were classified as pathogenic, likely pathogenic, uncertain significance (VUS), likely benign, or benign. As per ACMG classification, the variants of *SH2B3* and *KMT2D* may be VUS, but the variants of *BCORL1* may be likely benign. There is no sufficient literature to support pathological significance, and with further research, these variant sites may have relevant pathological significance in the future.

**Table 1:** The mutations of pediatric AML in this study.

Gene	NM	Allele change	Mutation	VAF (%)
*GNB1*	NM_002074	c.346G > A	G116S	46.6
*BCORL1*	NM_021946	c.2632A > G	S878G	99.95
*SH2B3*	NM_005475	c.1606G > A	A536T	51.17
*KMT2D*	NM_003482	c.11124C > G	S3708R	48.95

### Treatment and outcome

The patient presented with pneumonia and was administered intravenous latamoxef sodium (40 mg/kg/day, every 12 h) and subsequently meropenem (20 mg/kg/day, every 8 h) for anti-infection therapies. Several other symptomatic supportive treatments were used to prevent hyperuricemia and edema. Because the relatives disagreed with the combination chemotherapy, the patient did not receive medication. After 26 days of hospitalization, the patient eventually died of respiratory failure.

### Literature review

**Figure 2:**
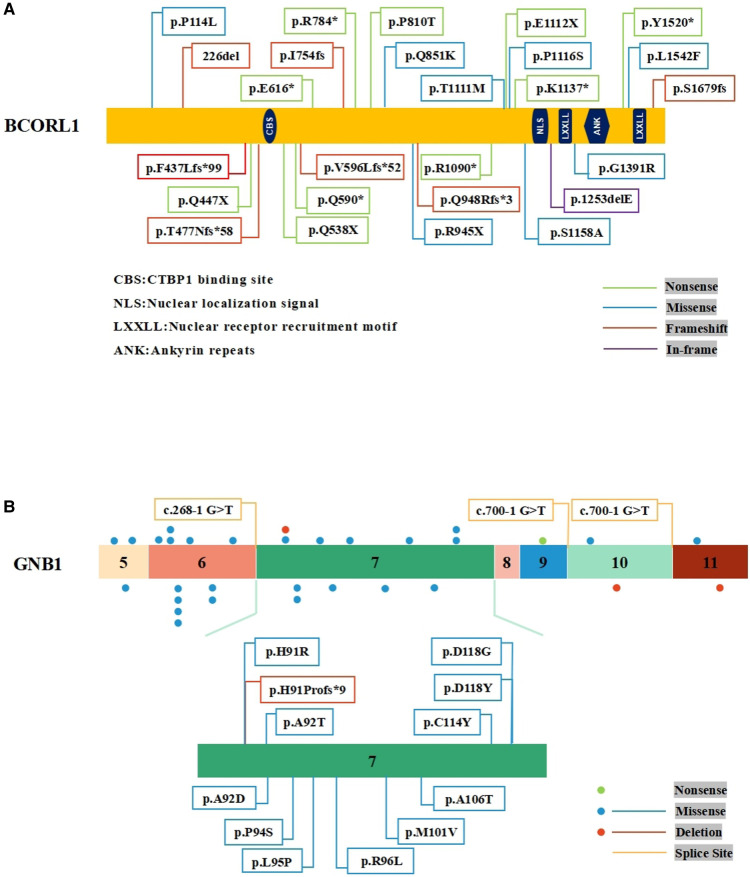
Schematic representation of *BCORL1* and *GNB1* mutations. (**A**) The mutations of *BCORL1* in AML. (**B**) The mutations in Exon 7 of the *GNB1* gene.

PubMed database was searched with keywords including acute myeloid leukemia, *BCORL1*, *GNB1*, *SH2B3,* and *KMT2D*. It has been reported that 26 different types of *BCORL1* mutations were described (2, 10–13) (Figure 2A). Pediatric AML with *BCORL1* mutations is very rare. To our knowledge, only six cases have been reported in the literature (2, 13). Most of them had complex chromosomal karyotypes with diverse chromosomal abnormalities (Table 2). Only one patient with *SH2B3* mutation (p.S417*) in SH2 domains was reported in a cohort of 1,540 patients with AML till now (14). *GNB1* mutations were present in 3 (1.9%) of 157 cases of myelodysplastic syndrome (MDS) or secondary AML (15). However, *KMTD2* mutations have not been described previously in AML.

**Table 2:** Characteristics of pediatric AML with gene mutations in *BCORL1*.

Case	Age (years)	Sex	FAB	Allele change	*BCORL1* mutation	Type of mutation	Karyotype	Prognosis
1^[2]^	6	F	M2	C > T	P1116S	Missense	45,X,−X,t(8;21)(q22;q22)[20]	Alive
2^[2]^	8	M	M2	C > T	P114L	Missense	46,XY,t(8;21)(q22;q22)[20]	Alive
3^[2]^	10	F	M0	/-TC	226del	Frameshift deletion	46,XX,t(8;12)(q11.2; p11.2)	Alive
4^[2]^	14	M	M2	C > T	R945X	Nonsense	45,X,-Y,t(8;21)(q22;q22)[19]/46, XY[1]	Alive
5^[2]^	15	M	M5b	C > T	Q447X	Nonsense	47,X,−Y,add(3)(q11.2),+6,add(6)(p21) × 2,+7,del(8)(q24),der(8)t(1;8)(q11;q24), del(11)(q?),add(17)(p11.2)[7]/48,sl,+22[6]/47,sl,−14,+mar1[2]	Death
6^[13]^	17	M	M1	NA	p.G158*(Stop)	Nonsense	46,XX[26]	Death

M, male; F, female; NA, not available.

## Discussion

AML is a heterogeneous disease characterized by the presence of different collaborating cytogenetic and molecular aberrations. Cytogenetic analysis had implicated several recurrent chromosomal structural abnormalities for pediatric AML pathogenesis, including 11q23 (KMT2A) rearrangements (frequency: 20%), t(8;21) (q22; q22) (frequency: 10%–12%), inv (16) (p13.1q22) or t(16;16) (p13.1;q22) (frequency: 10%), t(8;16) (p11;p13) (frequency:10%), t(15;17) (q24.1;q21.2) (frequency: 5%–10%), and t(6;9) (p22;q34) (frequency:1.2%–4%) (3). These acquired genetic abnormalities played an essential role in the pathogenesis of AML. Here, we described a pediatric AML with 48, XY, +13, +19[12]/48, idem, del(p12)[8]. In addition, sequencing technologies detected various mutations, including *BCORL1*, *GNB1*, *SH2B3,* and *KMT2D*. Although some of these mutations have rarely been reported in AML previously, mutations in the same region have been detected in patients with malignancies.

*BCORL1* (S878G) somatic mutation has been found in intracranial germ cell tumors (16), but *BCORL1* (S878G) germline mutation was reported in AML for the first time. To date, 26 different types of *BCORL1* mutations have been reported and characterized among 572 adult AML patients, which included cases with secondary leukemia and 287 pediatric patients, including 10 nonsense mutations, 8 missense mutations, 7 frameshift mutations, and 1 in-frame mutation (Figure 2A). To a certain extent, these mutations may truncate the encoded proteins. Further, *BCORL1* mutations were detected in the AML-193, SKM1, and OCI-AML5 cell lines, and all *BCORL1* mutations in cell lines truncated the encoded protein as a result of nonsense mutations, splice site mutations, or out-of-frame insertions or deletions (10). Although not resulting in clearly diminished *BCORL1* mRNA expression levels, these disruptive events were predicted to encode truncated proteins lacking the last C-terminal LXXLL nuclear receptor recruitment motif. This result not only implicates *BCORL1* mutations in the pathogenesis of AML but also demonstrates that *BCORL1* is a tumor suppressor gene inactivated by these mutations.

Mutations involving *BCORL1* and other mutations were associated with adverse outcomes in AML. One pediatric AML had a normal karyotype but carried *BCORL1* mutation [p.G158*(Stop)] and *RUNX1* mutation and eventually died (13). Terada et al. reported that *BCORL1* mutations had a significant positive correlation with *RUNX1* mutations, which were thought to indicate an unfavorable prognosis in AML (11). In fact, *BCORL1*-mutated AML had concomitant other mutations, including *KRAS*, *NRAS*, *IDH1* and *IDH2*, *DNMT3A*, *RUNX1,* and *FLT3-ITD,* and were mutually exclusive with *TP53, CEBPA,* or *NPM1* mutations (10). However, due to the low number of co-mutations in *BCORL1*-mutated AML, additional studies are necessary to confirm prognostic information.

Mutations in *GNB1* have been associated with myeloid and B-cell malignancies. Yoda et al. reported that *GNB1* K57 mutations were in myeloid neoplasms, while most of the *GNB1* I80 mutations were in B-cell neoplasms (17). *GNB1* (G116S) somatic mutation has been reported in acute lymphoblastic B-cell leukemia (18), but *GNB1* (G116S) mutation has not been described in AML. Moreover, an activating mutation of *GNB1* (p.K89M) in ETV6-ABL1-positive leukemic cells led to a poor response to tyrosine kinase inhibitors therapy (19). These results suggest that *GNB1* mutations may be closely related to hematological tumorigenesis. According to the published results, nearly 30 *GNB1* mutation carriers have been identified so far. Most of the *GNB1* mutations were located in Exon 6 and Exon 7, indicating the presence of a mutational hotspot. There have been found 12 mutations in Exon 7 of *GNB1* (5, 20) (Figure 2B). Functional studies demonstrated that specific mutations affected the *GNB1* residues that mediated interactions with both G proteins subunits and effector proteins. For example, the *GNB1* mutation (p.M101V) affects a G*β* residue found important for (1) activation of adenylyl cyclase 2, (2) inhibition of calcium channels, (3) activation of phospholipase C-*β*2, and (4) G-protein-coupled receptors (21). In addition, *GNB1* mutations activated AKT/mTOR signaling in human leukemia cells, and treatment with PI3K/mTOR inhibitor (BEZ235) suppressed PI3K-mTOR signaling and improved the survival of mice with mutant *GNB1*-induced leukemia (17). AML patients harbored leukemia cells that displayed dysregulation of the PI3K-Akt-mTOR signaling, and activation of this pathway also had a prognostic impact (22). Upregulated and dysregulated cytokine signaling is a major mechanism in the pathogenesis of multiple hematopoietic malignancies. Although the relevance remains unclear, we cautiously speculate that this paradigm might be applicable to *GNB1*-related AML.

The *SH2B3* gene was found on chromosome 12q24.12, and the majority of *SH2B3* mutations was missense mutations (23). Several *SH2B3* mutations were identified in patients with MPNs or the related idiopathic erythrocytosis (W364X, S370C, L390W, R392Q, S394C, E395K, E400K, V402M, R415H, R415C, R425C, M437I, and I446V) and in patients with B-cell precursor ALL (R392W, R397G, and Q427P) (24). The mutational loss of function of *SH2B3* in hematopoietic cells released its inhibitory activity against the activated tyrosine kinase receptors and its downstream JAK-STAT pathway, resulting in the enhanced proliferative capacity of hematopoietic cells. Wu et al. reported that *SH2B3* (A536T) mutation was found in chronic myeloid leukemia (25). *SH2B3* (A536T) mutation led to the substitution of alanine for threonine at position 536, which has not been reported in AML. The mechanisms of *SH2B3* mutations in AML are unknown, and the potential effects require further investigation.

The *KMT2D* gene was mutated and deleted in many different types of cancer, including leukemia, bladder, lung, liver, prostate, breast, ovarian, gastric, pancreatic, renal, and colorectal cancers (26). Considering the effect of *KMT2D* on gene expression regulation, as well as its non-enzyme-dependent function, mutations of *KMT2D* may contribute to aberrant gene expression programs, thereby driving tumor malignancies. *KMT2D* had two frequently occurring genomic alterations: nonsense and frameshift mutations, which may generate truncated KMT2D proteins with a deficient methyltransferase activity (7, 27). Moreover, Kantidakis et al. pointed out that *KMT2D* mutation resulted in transcription stress and genome instability as a strong driver in tumorigenesis (28). However, *KMTD2* (S3708R) mutation has not been described previously, and the significance and role of *KMT2D* mutations were not determined in AML.

## Conclusion

To the best of our knowledge, this is the first reported case of pediatric AML with rare *BCORL1*, *GNB1*, *SH2B3,* and *KMT2D* mutations. This case highlights the possibility of an increased risk of hematological malignancies in individuals with co-occurring mutations, but the hierarchy and mechanism are poorly understood. Compared with the general population, this is an interesting study due to the rarity of this form and the resulting difficulty in finding patients to conduct further studies on. This paper aims to provide an opportunity to broaden the understanding available of this rare form of AML.

## Data Availability

The original contributions presented in the study are included in the article/Supplementary Material, further inquiries can be directed to the corresponding author/s.
